# Quality Assurance for Stereotactic Body Radiation Therapy for Gynecologic Malignancies

**DOI:** 10.7759/cureus.53470

**Published:** 2024-02-02

**Authors:** Samuel N Andersen, Mark D Bonnen, Michelle S Ludwig, Shraddha M Dalwadi

**Affiliations:** 1 Radiation Oncology, University of Texas Health San Antonio MD Anderson Cancer Center, San Antonio, USA; 2 Radiation Oncology, Baylor College of Medicine, Houston, USA

**Keywords:** radiation for gynecologic malignancies, sbrt boost, sbrt quality control, inoperable uterine cancer, inoperable cervical cancer, uterine cancer, cervical cancer, stereotactic body radiation therapy

## Abstract

The use of stereotactic body radiation therapy (SBRT) is not well studied or reported in the treatment of gynecologic malignancies, despite its success in the definitive management of other cancer sites. This report describes a rigorous quality assurance process for patients to undergo dose escalation to the pelvis via stereotactic photon beam irradiation. Patients who receive SBRT must be ineligible for conventional brachytherapy boost and undergo comprehensive informed consent. Fiducial placement, bowel prep, Foley catheter placement with standardized bladder filling, computerized tomography (CT) simulation with whole-body immobilization, magnetic resonance imaging (MRI)-assisted target delineation, planning aims based on the established brachytherapy literature, and physics consultation for SBRT plan optimization are necessary. Prior to each fraction, the simulation position is reproduced and verified with on-table cone beam CT, and the position is maintained with whole-body immobilization. Following treatment, the treating physician is active in survivorship and toxicity management. Gynecologic SBRT is an ongoing area of study, and preliminary successes in delivering high-quality stereotactic dose escalation suggest prospective investigation is warranted. By adhering to strict quality control measures and following a pre-defined best standard of practice, patients with gynecologic malignancies who are ineligible for traditional brachytherapy procedures can be safely treated with SBRT.

## Introduction

Identifying the best local therapy for locally advanced, inoperable, recurrent, and oligometastatic gynecologic malignancies is often challenging [1, 2]. The use of stereotactic body radiation therapy (SBRT) in the treatment of gynecologic malignancies has not been studied in a prospective clinical trial, but its use in this setting has been reported on in multiple retrospective series, as summarized by Ladbury et al. [3]. Stereotactic body radiation therapy is a highly conformal, ultra-hypofractionated form of external beam radiotherapy [4]. The sharp fall-off of the high-dose region allows dose escalation while sparing nearby normal structures, necessitating the use of accurate image guidance and strict patient immobilization [4]. Despite the clear use of SBRT in gynecologic malignancies documented in an American population-based study, little data exists regarding the technical considerations involved with this technique [5].

While most women are candidates for preferred management (i.e., brachytherapy or surgical resection), a population of patients who are not suitable candidates for these treatments does exist. Without a high level of evidence to guide management, considerable time and effort have been placed into outlining the following techniques to administer gynecologic SBRT. This approach has yielded relatively favorable toxicity, local control, and survival outcomes, with five-year follow-up data published [6].

## Technical report

**Figure 1:**
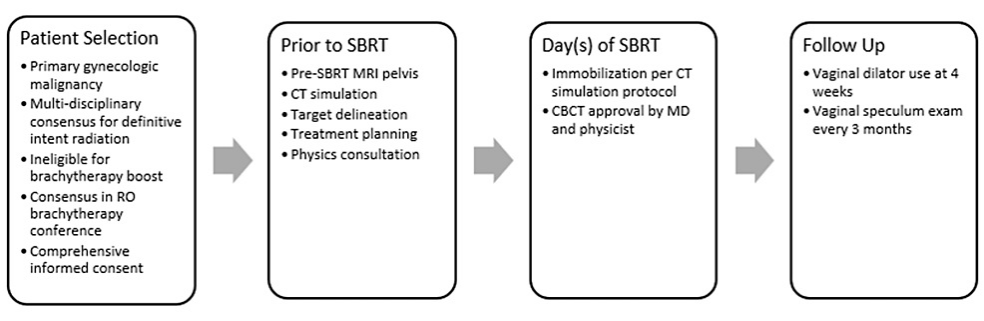
Gynecologic SBRT quality assurance process SBRT: stereotactic body radiation therapy; MRI: magnetic resonance imaging; RO: radiation oncology; CBCT: cone beam computed tomography

A rigorous quality assurance process for gynecologic SBRT is described in Figure 1. 

Patient selection 

Candidates must have a tissue diagnosis of primary gynecologic malignancy with multidisciplinary consensus for definitive management. All patients should be discussed among colleagues at the departmental pre-treatment brachytherapy conference, where brachytherapy options are given first consideration. 

**Figure 2:**
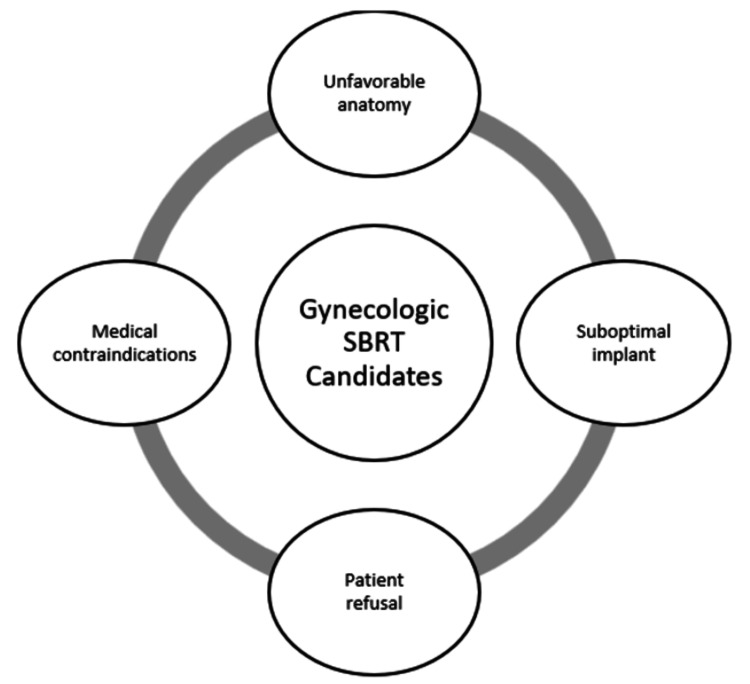
Possible candidates for gynecologic SBRT SBRT: stereotactic body radiation therapy

Patients offered SBRT typically fall into one of four categories (Figure 2).

The following were the possible candidates for gynecologic offering SBRT: 1) patients with unfavorable anatomy, including disease measuring greater than 22 centimeters in the craniocaudal direction, precluding total target coverage with brachytherapy; 2) patients for whom tandem placement is impossible due to cervical os obliteration by tumor and/or treatment effect after attempted implantation; 3) patients with medical contraindications precluding the ability to undergo general anesthesia, refractory bleeding disorders, and a high risk of preoperative death as determined by anesthesiology. 4) patients who refuse an invasive procedure.

Patients must have an Eastern Cooperative Oncology Group (ECOG) performance status of less than or equal to three with no pelvic surgeries at least six weeks prior to SBRT. The last dose of cytotoxic chemotherapy must be given at least five days prior to the first fraction of SBRT. Prior to SBRT, three-dimensional volumetric gynecologic protocol magnetic resonance imaging (MRI) must be obtained to evaluate the extent of disease and invasion into nearby structures; images are reviewed with diagnostic radiology staff [6-8]. Finally, candidates need to undergo voluntary, comprehensive, and informed consent, with special attention given to potential side effects and options (Table 1).

**Table 1:** Informed consent process for gynecologic SBRT SBRT: stereotactic body radiation therapy

Possible side effects of gynecologic SBRT
Fistula
Bowel injury/rupture
Urinary tract stenosis/stricture
Chronic cystitis
Arteriovenous malformations
Pelvic adhesions limiting future surgery

Simulation protocol

Magnetic resonance imaging-compatible fiducials are placed at the nine, 12, and three o’clock positions within the cervix or cervical tumor during an in-clinic vaginal speculum exam for cervical cancer patients. For inoperable endometrial cancer patients, four fiducials would optimally be placed within the uterus during a fundoscopic exam by the treating gynecologic oncologist; however, cervical fiducials may still be useful in tandem with pre-treatment MRI and computed tomography (CT)-based organ delineation. A 2 mm or smaller slice thickness CT simulation is performed with the patient supine and arms down. Bladder filling is facilitated by catheter placement and filling to a prespecified amount with normal saline for improved reproducibility; this may also aid in minimizing the dose to the small bowel. An empty rectum is achieved with a dedicated bowel preparation regimen, per institutional experience. A vaginal cylinder may be inserted to displace redundant tissue on a case-by-case basis. The patient’s position is secured with a whole-body vacuum-lock system with a poncho. The radiation oncologist and physicist must approve the immobilization setup and the simulation scan.

Target delineation 

The simulation CT scan is fused with the pre-SBRT volumetric MRI for target delineation prior to contouring (Table 2).

**Table 2:** Target delineation for gynecologic SBRT SBRT: stereotactic body radiation therapy; EBRT: external beam radiation therapy (conventional dosing, i.e., typically 2Gy or less per fraction); MRI: magnetic resonance imaging; GTV: gross tumor volume; CTV: clinical target volume; IR-CTV: intermediate-risk clinical target volume; HR-CTV: high-risk clinical target volume SBRT

Structure	Guidelines
Target	
IR-CTV	All pre-treatment pelvic disease not dose-escalated beyond 55Gy (*Only applicable in cases of SBRT in lieu of brachytherapy*)
HR-CTV	All residual disease evident on post-pelvic EBRT MRI + entire cervix if the patient had cervical involvement initially (*For endometrial cancer patients, GTV can equal HR-CTV if no cervical involvement is present*)
GTV-residual	All residual disease evident on post-pelvic EBRT MRI
Organs at risk	
Bladder	Outline the entire organ including the bladder wall and bladder neck
Rectum	Outline the rectum from the anal verge to the recto-sigmoid junction including the rectal wall; extends exactly 12cm cranio-caudally
Sigmoid	Must extend beyond 12cm from the anal verge to the iliac fossa
Small bowel	Individual remaining bowel loops must be outlined as a conservative bowel bag; must include the entire pelvis and extend 3cm above the most superior axial CTV slice
Bone marrow	Outline the intramedullary space of the bony pelvis
Femoral heads	Both femoral head and neck to the level of the lesser trochanter

The bladder, rectum, sigmoid, bone marrow, and small bowel are contoured as organs at risk. A high-risk clinical target volume (HR-CTV) is delineated and includes the gross tumor volume (GTV) as determined by pre-SBRT MRI and physical examination. For cervical malignancies and uterine cancer with cervical involvement, the entire cervix is included in the HR-CTV as per standard MRI-guided brachytherapy practices [8]. A planning target volume (PTV) margin of 3 mm circumferentially and 1 mm posteriorly is utilized to account for daily patient positioning changes.

An intermediate-risk CTV (IR-CTV) is defined as the extent of disease prior to the initiation of oncologic management, delineated using the fusion of the initial diagnostic MRI with the simulation CT as per standard brachytherapy practices.

Treatment planning 

**Figure 3:**
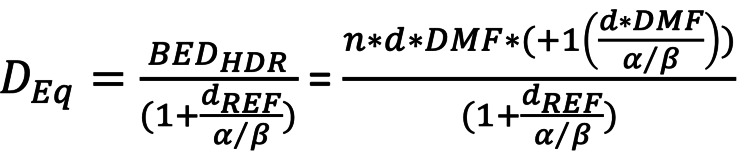
Cumulative dose calculation formula by Nag S and Gupta N Reference: Nag S and Gupta N, 2000 [8] Deq: equivalent dose; BED: biologically effective dose; HDR: high dose rate therapy (classically brachytherapy); d_REF: reference dose per fraction for a conventionally fractionated external beam treatment (assumed to be 2Gy); alpha/beta: property of a cell line conveying its response to radiation dose and fractionation; n: number of fractions; d: dose per fraction; DMF: dose modifying factor

Similar to brachytherapy doses, cumulative dose calculations are derived using Nag and Gupta's formula, assuming a tumor alpha/beta ratio of 10 and a normal tissue alpha/beta of three (Figure 3) [9].

No dose-modifying factor (DMF) is used, allowing for a conservative estimate of the total dose [9]. Relevant cumulative doses in 2Gy per fraction equivalents (EQD2) from the initial external beam treatment and the SBRT boost are documented for the targets and organs at risk.

The planning aims for SBRT in lieu of brachytherapy are described in Table 3, adapted from the EMBRACE II study [8].

**Table 3:** Cumulative dose plan evaluation goals adapted from the EMBRACE II study Reference: Pötter R, et al., 2018 [8] IR-CTV D98: dose delivered to 98% of the intermediate-risk clinical target volume; HR-CTV D90: dose delivered to 90% of the high-risk clinical target volume; HR-CTV D98: dose delivered to 98% of the intermediate-risk clinical target volume; GTV-residual D98: dose delivered to 98% of the gross tumor volume

Description	Hard constraints	Planning aims
Target		
IR-CTV D98		>60Gy
HR-CTV D90	>85Gy	90-95Gy
HR-CTV D98		>75Gy
GTV-residual D98	>90Gy	>95Gy
Organs at risk		
Bladder dose to 2cc	<90Gy	Planning aim: <80Gy
Rectum dose to 2cc	<75Gy	Planning aim: <70Gy
Sigmoid dose to 2cc	<75Gy	Planning aim: <70Gy
Bowel dose to 2cc	<70Gy	Planning aim: <65Gy

The typical fractionation schema is 25-35Gy in four to five fractions to the HR-CTV, but this is adjusted based on anatomical considerations and organ-at-risk (OAR) tolerances. For example, due to the common use of intensity-modulated radiotherapy with simultaneous and sequential nodal boosts for gynecologic patients, a thorough audit of the external beam plan for hot and cold spots is necessary to account for potential excessive dose overlap or areas requiring more coverage. The prescription is converted to an equivalent dose in 2Gy per fraction (EQD2), assuming an alpha/beta ratio of 10 for the tumor and an alpha/beta ratio of three for the normal tissues, to assess the summation of the EBRT and SBRT plans when evaluating the total dose received by the GTV and OARs. Cumulative doses beyond hard constraints are generally not allowed, as exceeding hard constraints was noted in EMBRACE II to correlate very closely with increased toxicity; HR-CTV coverage may be limited due to priority being placed on normal tissue constraints. Great care is taken to mimic brachytherapy dosing as closely as possible, including allowance for central areas of high dosage within the target with rapid dose fall-off outside of the target adjacent to normal tissues.

Stereotactic body radiation therapy plan quality is primarily evaluated with the ultimate goal of mirroring brachytherapy dose falloff. This includes slice-by-slice evaluation with close attention to adjacent OARs. In addition, technical parameters are adapted from Radiation Therapy Oncology Group (RTOG) 0813 (Table 4) [10].

**Table 4:** SBRT quality evaluation adapted from RTOG 0813 Reference: Bezjak A, et al., 2019 [10] SBRT: Stereotactic body radiation therapy; RTOG: Radiation Therapy Oncology Group; PTV: planning target volume

	Criteria	Goal	
Coverage	Percentage of PTV receiving 100% of the prescription dose	>95%	
	Percentage of PTV receiving 90% of the prescription dose	>99%	
Heterogeneity	Ratio of the prescription dose over the maximum dose	60-90%	
Conformality index	Ratio of the volume receiving 100% of the prescription dose to the PTV	No deviation	Minor deviation
		<1.2	<1.5
High-dose spillage	Volume receiving 105% of the prescription dose or more outside of the PTV	<20% of the PTV volume	

If two dose levels are used (IR-CTV and HR-CTV), HR-CTV metrics are used when determining the quality of SBRT. Target coverage is scrutinized closely, with the goal of achieving a V100 greater than or equal to 95% and a V90 greater than or equal to 99%. Heterogeneity must be between 60% and 90%. High-dose spillage cannot extend beyond 20% of the PTV volume. Additionally, high-dose spillage cannot enter gastrointestinal OARs.

Day of treatment

Treatments are generally fractionated over five treatments delivered twice weekly or every other day (to minimize total "package time" of treatment delivery) using a stereotactic-enabled linear accelerator with at least 5mm leaf collimation and ideally hexapod treatment couch capability. On each day of SBRT, the patient is brought to the treatment room and placed in a treatment position, mimicking a CT simulation. Image guidance with a cone-beam CT must be approved by both a physician and a physicist. Fiducials are used for guidance to line up the cone beam CT scans. Occasionally, a significant treatment response is seen midway through the SBRT regimen and will require adaptive re-planning.

Documentation and follow-up

A physics consult is generated that includes documentation of cumulative dose calculations, planning aims, and SBRT quality by dosimetry and physics. Slice-by-slice images of the final treatment plan and a comprehensive dose volume histogram are included in the patient’s chart. A standard end-of-treatment summary is generated as well that describes the total dose of radiation delivered via both standard pelvic fields and SBRT boost.

Patients are given patient education upon discharge regarding precautions for returning to the emergency department. Vaginal dilator use to prevent vaginal stenosis is encouraged beginning four weeks after treatment. Patients are generally followed at three-month intervals with a vaginal speculum exam and imaging as indicated.

## Discussion

The investigation of SBRT extends beyond early-stage lung cancer to the management of other oncologic sites and even benign disorders. For example, SBRT as monotherapy and boost is now considered a standard option in prostate cancer [11]. In gynecologic malignancy, the ability of SBRT to mimic brachytherapy dose distributions and evidence of its use in practice is well documented [3-4, 12-13]. This paper represents the first effort to publish a standard quality assurance process for safe and effective gynecologic SBRT.

A phase II prospective clinical trial evaluating gynecologic SBRT as a boost after pelvic chemoradiation treated 42 patients and reported a 78.6% local control rate at a five-year median follow-up without any grade 3 or greater urinary or bowel toxicity [14]. Another phase II study of SBRT for gynecologic malignancy dose-escalation reported a two-year cumulative rate of grade 3 or greater toxicity in 26.7% of patients, primarily consisting of rectal ulcer/recto-vaginal fistulas. Notably, toxicity was more common in patients with larger tumor volumes (median volume of 225cc), suggesting extra attention to OARs is warranted in these patients [15]. Notably, the Mantz trial limited patient tumor size to less than 125cc, which may have allowed for more favorable dosimetry and correlated with the lack of grade 3 toxicity [14]. In the Albuquerque paper, fiducials, pre-treatment bowel preparation, and a Foley catheter for bladder fill were used in a similar fashion to the recommendations of this present technical report; a notable difference from this report was the goal of prescribing dose homogeneously, as opposed to mimicking typical SBRT plans, which allow for greater heterogeneity with a high dose centrally and a more significant dose falloff approaching normal tissues [15].

While SBRT for gynecologic malignancies is not a routine approach, relatively high rates of local control and low rates of cancer-specific mortality in uterine and cervical cancer patients treated with this protocol have been previously reported, with minimal grade 3 or higher toxicity [6-7]. When utilizing a methodical approach to quality assurance, prioritizing normal tissue dose constraints, and placing emphasis on patient safety, gynecologic SBRT appears to be a viable option when traditional brachytherapy boost approaches are not feasible. The process of delivering gynecologic SBRT boost requires caution from start (careful collaborative patient selection) to finish (treatment delivery).

## Conclusions

Patients with locally advanced, inoperable, recurrent, or oligometastatic gynecologic malignancies ineligible for brachytherapy can be treated with a variety of different methods, as no standard of care currently exists. The details outlined in this report regarding a strict quality assurance process for fractionated pelvic SBRT may enable a more widespread and standardized application of this approach for a well-selected patient population. Further evaluation of this ubiquitous technology in the gynecologic setting should be undertaken.
